# Randomized clinical study comparing active heating methods for prevention of intraoperative hypothermia in gastroenterology*

**DOI:** 10.1590/1518-8345.2589.3103

**Published:** 2019-01-14

**Authors:** Regina Maria da Silva Feu Santos, Ilka de Fatima Santana Ferreira Boin, Cristina Aparecida Arivabene Caruy, Eliane de Araújo Cintra, Nathalia Agostini Torres, Hebert Nogueira Duarte

**Affiliations:** 1 Universidade Estadual de Campinas, Hospital de Clínicas, Campinas, SP, Brazil.; 2 Universidade Estadual de Campinas, Faculdade de Ciências Médicas, Campinas, SP, Brazil.; 3 Hospital Sarah Kubitschek, Brasília, DF, Brazil.

**Keywords:** Hypothermia, Nursing, Perioperative Period, Body Temperature Regulation, Body Temperature, Equipment and Supplies, Hipotermia, Enfermagem, Período Perioperatório, Regulação da Temperatura Corporal, Temperatura do Corpo, Equipamentos e Provisões, Hipotermia, Enfermería, Período Perioperatorio, Regulación de la Temperatura Corporal, Temperatura del Cuerpo, Equipos y Provisiones

## Abstract

**Objective:**

to compare the efficacy of three active heating methods in the prevention of intraoperative hypothermia in open gastroenterological surgeries.

**Method:**

randomized clinical trial with a sample of 75 patients, whose initial body temperature measured by a tympanic thermometer. Esophageal temperature <36ºC was considered hypothermic. Patients were divided into three groups using: thermal mattress, underbody forced-air warming blanket and heated infusion system. The tympanic and esophageal temperatures were measured at different times of the intraoperative period, but the temperature considered gold standard was the esophageal. To evaluate the homogeneity of the groups, we used chi-square test (categorical variables). In the comparison of temperature measurements over time, the analysis of variance (ANOVA) and the contrast profile test were used for the difference in temperature between the times. The non-parametric Kruskal-Wallis test was used to compare the three groups. The level of significance was 5%.

**Results:**

regarding the studied variables, the groups were not homogeneous as to the categorical variable sex. All patients presented hypothermia during the intraoperative period (p> 0.05).

**Conclusion:**

there was no significant difference between the heating methods in the prevention of intraoperative hypothermia. REBEC - Brazilian Registry of Clinical Trials (RBR- no. 52shjp).

## RESUMO

**Objetivo:**

comparar a eficácia de três métodos de aquecimento ativo na prevenção da hipotermia intraoperatória em cirurgias gastroenterológicas por via aberta.

**Método:**

ensaio clínico randomizado com amostra de 75 pacientes, com temperatura corpórea inicial mensurada por termômetro timpânico. Considerou-se hipotermia a temperatura esofágica <36ºC. Foram distribuídos em três grupos: colchão térmico, manta de aquecimento de ar forçado *Underbody* e sistema de infusão aquecida. As temperaturas timpânica e esofágica foram aferidas em diferentes momentos do intraoperatório, mas a temperatura considerada padrão ouro foi a esofágica. Para avaliar a homogeneidade dos grupos, utilizou-se o teste qui-quadrado (variáveis categóricas). Na comparação das medidas de temperatura ao longo do tempo, a análise de variância (ANOVA) e teste de perfil de contraste para a diferença das temperaturas entre os tempos. Para comparação dos três grupos, usou-se o teste não paramétrico de Kruskal-Wallis. O nível de significância foi de 5%.

**Resultados:**

em relação às variáveis estudadas, os grupos não foram homogêneos quanto à variável categórica sexo. Todos os pacientes apresentaram hipotermia no período intraoperatório (p > 0,05).

**Conclusão:**

não houve diferença significativa entre os métodos de aquecimento na prevenção da hipotermia intraoperatória. REBEC - Registro Brasileiro de Ensaios Clínicos (RBR- nº52shjp).

## RESUMEN

**Objetivo:**

comparar la eficacia de tres métodos de calentamiento activo en la prevención de la hipotermia intraoperatoria en cirugías gastroenterológicas por vía abierta.

**Método:**

ensayo clínico aleatorizado con muestra de 75 pacientes, con temperatura corpórea inicial, medida por termómetro timpánico. Se consideró hipotermia la temperatura esofágica de <36ºC. Fueron distribuidos en tres grupos: colchón térmico, manta de calentamiento de aire forzado *Underbody* y sistema de infusión calentada. Las temperaturas timpánica y esofágica fueron medidas en diferentes momentos del intraoperatorio, pero la temperatura considerada estándar oro fue la esofágica. Para evaluar la homogeneidad de los grupos utilizamos el test Chi-cuadrado (variables categóricas). En la comparación de las medidas de temperatura a lo largo del tiempo, el análisis de variancia (ANOVA) y el test de perfil de contraste se usaron para la diferencia de las temperaturas entre los tiempos. Para comparación de los tres grupos se usó el test no-paramétrico de Kruskal-Wallis. El nivel de significancia fue de 5%.

**Resultados:**

en relación a las variables estudiadas, los grupos no fueron homogéneos en la variable categórica del sexo. Todos los pacientes presentaron hipotermia en el período intraoperatorio (p > 0,05).

**Conclusión:**

no hubo diferencia significativa entre los métodos de calentamiento en la prevención de la hipotermia intraoperatoria. REBEC - Registro Brasilero de Ensayos Clínicos (RBR- nº52shjp).

## Introduction 

The body loses heat from four mechanisms: radiation, conduction, convection and evaporation. During the anesthetic procedure, hypothermia (body temperature <36ºC) can occur due to redistribution of heat from the central compartment to the periphery in view of the use of anesthetic drugs or the receipt of large volumes of intravenous fluids and irrigation^1^ ^-^ ^3^. 

Hypothermia may cause increased blood pressure, heart rate and intracranial pressure, in addition to arrhythmias, coagulopathy, infection, increased peripheral vascular resistance and reduced metabolism, among others. The body produces tremors which is 50% to 100% of heat production in adults^4^ ^-^ ^6^. Approximately 70% of the patients present hypothermia during the intraoperative period, which can be classified as mild (32 to 35ºC), moderate (28 to 32ºC) or severe (<28ºC)^5^ ^-^ ^7^. In operating rooms, the room temperature can vary between 18 and 23°C, providing a pleasant temperature to the team and avoiding the multiplication of microorganisms, since it is a relevant factor for heat loss^8^.

The forced air circulation device consists of a temperature management unit, which comprises a heat generator^9^. In a comparative study, the authors showed that the use of the thermal mattress was more efficient than the warming blanket in the prevention of hypothermia in patients submitted to open abdominal surgery^10^. In an investigation comparing conduction heating (thermal mattress) alone and conduction associated with convection (thermal mattress and warming blanket), the authors concluded that there was no reduction in the incidence of complaints of cold and postoperative tremors^11^. There is a limited number of national studies on the understanding of hypothermia, as well as comparing effective methods for the prevention and treatment of this complication. Intraoperative inadvertent hypothermia can cause several complications. Thus, its prevention is important, since we can guarantee patient safety by preventing the risks^11^. In this way, we intend to investigate the effectiveness of heating methods. This study may provide subsidies for the planning of intraoperative nursing care, as well as for planning the acquisition of resources for the prevention of hypothermia.

The objective of the study was to compare the efficacy of three active heating methods in the prevention of intraoperative hypothermia in open gastroenterological surgeries. 

## Method

The study design was a randomized clinical trial, developed at the surgical center of a public university hospital in the interior of the state of São Paulo. This work was approved by the Research Ethics Committee of the Faculty of Medical Sciences, Unicamp (CEP 1269/2011), (REBEC -RBR- no. 52shjp). 

Data collection took place from October 2012 to July 2015 in patients submitted to gastroenterological surgeries, of both sexes, aged 18 years or older, with physical status Ps1-Ps4 according to ASA-PS (American Society of Anesthesiologists - Physical State)^12^, being submitted to general anesthesia, according to the routine procedure of the HC/Unicamp Anesthesia Service. 

Exclusion criteria were patients with a body mass index (BMI) <20> 30, age extremes, initial tympanic body temperature below 36° C or equal to or greater than 38° C, transfusion of more than two bags of blood components, volume replacement greater than 30% of that recommended by the local anesthesia service (15 ml/kg weight at 1^st^ hour and 10 ml/kg, subsequent weight) and patients in whom surgical resectability proposed in the study objective was not performed. The sample size was determined with an alpha sample error of 5% for a 95% confidence level and a 20% beta error, indicating the need for 24 patients/group for a difference in temperature greater than 0.1 between groups. The randomization procedure was performed in 100 patients due to possible losses during the surgical process. After signing the Informed Consent Form, the heating methods described were put inside a brown, opaque and sealed envelope, and drawn. The envelope was opened in the operating room (OR) before the anesthetic procedure. The study was masked because neither the anesthetists, nor the surgeons nor the operating room assistants knew which method had been drawn; only the researcher knew it.

At the reception of the patient in the surgical center, the tympanic temperature was monitored for exclusion and control so that the patients did not enter in surgery in a hypothermic state (<36Cº). 

All patients were submitted to preheating with a hot and forced air overlap blanket in the preparation room for 15 minutes before being conducted to the OR. The tympanic temperature of all patients was measured before and after preheating. The patients were covered with surgical drapes, leaving only the abdominal region for xifo-pubic incision. All patients who did not belong to the heated infusion group received liquids at room temperature. The esophageal temperature was measured at different moments of the intraoperative period. The temperature considered as the gold standard for statistical analysis of the effectiveness of the heating methods was the esophageal, since it was considered of greater precision^11^. Esophageal temperature monitoring was obtained with a sensor positioned at the transition from the hypopharynx to the esophagus. Temperature recording was performed on a multiparametric monitor, DPM7™ Mindray^®^ Display Screen/New Jersey, USA following the following order: after anesthetic induction, in the 1st, 2nd and 3rd hours, at the end of surgery and pre-extubation. 

The most accurate temperature is the central one, and the most reliable measurements are those performed on the tympanum, esophagus, nasopharynx and pulmonary artery^11^. 

The room temperature of the OR was monitored by the Minipa MT-242^®^ thermo-hygrometer Joinville/SC/Brazil and maintained at 22°C-24°C, following the guidance of the American Society of PeriAnesthesia Nurses (ASPAN)^13^. The sample was randomized into three groups. The first one is the thermal mattress group (GI, n = 33) using the *Gaymar Medi Therm MTA-4700 Hyper-Hypothermia System* ^®^ equipment, Orchard Park, NY/USA. The mattress was covered by a cotton sheet and regulated to the target temperature of 38 ± 0.5°C, keeping it connected from the patient›s entrance into the OR until their referral for anesthetic recovery. 

The second is the heated infusions group (GII n = 35), with the Ranger^TM^, *Irrigation fluid Warming system 247* 3M^®^ equipment, MN/USA. The Ranger heating system is designed to heat fluids and blood components and deliver them in the KVO system up to 30,000 ml/hour. It uses disposable devices that slide easily into the heating unit, being fitted in a single direction, free of connection errors. It has highly conductive aluminum heating plates that disperse heat evenly and immediately, presenting no risk of overheating and adapting to sudden changes in flow rates. It performs temperature monitoring four times per second with heating level adjustment, keeping the set temperature stable throughout the procedure. It has a visible and audible alarm system that ensures that the system operates effectively and safely in situations out of the normal temperature range. The device has an outlet temperature between 33°C and 41°C. It takes less than two minutes to warm up the set temperature to 41°C^14^.

The third group is the forced-air warming blanket group (GIII n = 32), with the *Bair Hugger System Temperature Management Unit - Model 775*, 3M^®^ equipment, California/USA. Patients were placed on the underbody forced-air warming blanket set at target temperature of 40-43° C, with effective heat transfer between the equipment and the blanket due to the high airflow, maximizing the patient’s body surface, allowing freedom for the surgical positioning and that the heating occurred since the beginning of the procedure^6^. This product was supplied by CEI (Comércio Exportação Importação de Materiais Médicos Ltda.). 

In this study, the independent variable was the heating method (GI, GII and GIII). The dependent variable was the central temperature variation. The continuous variables were age (years), body mass index (BMI in kg/m^2^), surgical time (in minutes), volume of blood components administered (in ml) and total volume of liquid administered (ml). The categorical variables were sex (male/female) and type of surgery. 

The chi-square test and the Kruskal-Wallis test were applied to assess the homogeneity of the groups. In order to compare the measurements of temperature over time, the analysis of variance (ANOVA) for repeated measures was used, followed by the contrast profile test to demonstrate the difference in temperature between the times (induction in 1st, 2nd, 3rd hours, end of surgery and extubation). The level of significance was 5%. The statistical program used was SAS system for Windows, version 9.4 (2012), Cary, NC, USA. 

## Results

**Figure 1:**
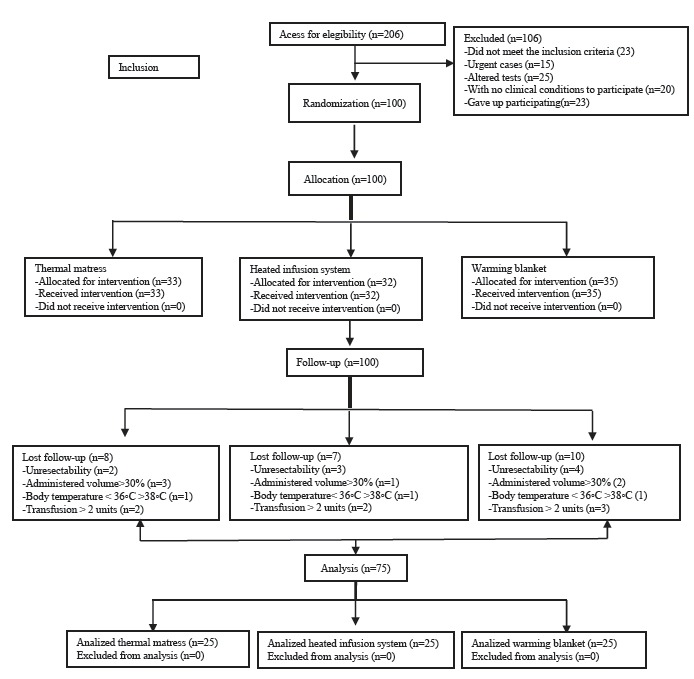
CONSORT flowchart applied to this study, Campinas, SP, Brazil, 2015

A total of 206 patients were eligible for the study, of whom 83 were excluded and 23 dropped out. So, 100 patients were randomized, as can be seen in Figure 1. 

Twenty-five patients were excluded (thermal mattress = 8, heated infusion = 10, blanket = 7) due to surgical unresectability (respectively 2, 3 and 4 patients); increased infusion volume (3, 1 and 2 patients); 36ºC < temperature > 38ºC (1, 1 and 3 patients) and transfusion >2 units (2, 2 and 3 patients). The study sample consisted of 75 patients and data from the continuous and categorical variables of the surgical procedure are shown below (Tables 1 and 2).

**Table 1:** Distribution of the continuous variables of the 75 patients studied. Campinas, SP, Brazil, 2015

Variables in Mean/Standard deviation	GI*-thermal mattress (n=25)	GII^†^-heated infusion system (n=25)	GIII^‡^-thermal blanket (n=25)	P^||^
Age (years)	50.5 ± 8.9	53.0 ± 9.0	52.8 ± 10.2	0.38
BMI^§^ (kg/m2)	22.9 ± 4.2	24.2 ± 4.5	24.0 ± 4.2	0.65
Surgical time (min)	278.2 ± 59.5	289.8 ± 90.1	297.8 ± 74.1	0.35
Transfusion (ml)	157.7 ± 239.5	183.6 ± 353.0	188.9 ± 254.3	0.89
Infusion of liquids (ml)	4610.3 ± 1027.4	4656.8 ± 1853.9	5036.6 ± 1657.1	0.50
Loss of liquids (ml)	3876.4 ± 1375.6	3442.0 ± 2061	3876.4 ± 1375.6	0.24

*G1-Group 1; †GII- Group 2; ‡GIII- Group 3; §BMI Body Mass Index; ||Kruskal-Wallis Test

**Table 2:** Distribution of the categorical variables of the 75 patients studied. Campinas, SP, Brazil, 2015

**Variables in** **Mean/Standard deviation**	GI*-thermal mattress **(n=25)**	**GII** ^**†**^ **-heated infusion system** **(n=25)**	**GIII** ^**‡**^ **-thermal blanket** **(n=25)**	P**
**Sex** **Male (n=42)** **Female (n=33)**	**11 (26.1%)** **14 (42.4%)**	**19 (45.2%)** **6 (18.1%)**	**12 (28.5%)** **13 (39.3%)**	**0.05**
**Types of surgeries** **GDP** ^**§**^ **Total Gastrectomy** **BDA** ^**||**^ **Others** ^**¶**^	**12 (48.0%)** **6 (24.0%)** **7 (28.0%)** **0**	**7 (28.0%)** **5 (20.0 %)** **6 (24.0%)** **7 (28.0%)**	**7 (28.0%)** **7 (28.0%)** **5 (20.0%)** **6 (24.0%)**	**0.16**

*GI-Group 1; †GII- Group 2; ‡GIII- Group 3; §GDP - Gastroduodenopancreatectomy; ||BDA bileodigestive anastomosis; ¶Other: pancreatectomy, exploratory laparotomy, gastroenteroanastomosis; **Kruskal-Wallis Test;

Homogeneity was observed between the groups, except for sex.

According to the analyzed variables, the majority of patients (56 = 74.6%) were classified by anesthesiologists as Ps 3 and the median surgical time was 285 (120-575) minutes. There was no significant difference between the groups studied, p = (0.23).

There was a significant difference in temperatures when we comparing the time between induction and the 1st, 2nd and 3rd hours, the end of surgery and extubation and between the 3rd hour and the end of surgery (p <0.0001), regardless of the analyzed group, which can be observed in Figure 1. However, there was no difference in temperature between the studied groups. P = (0.06).

**Figure 2:**
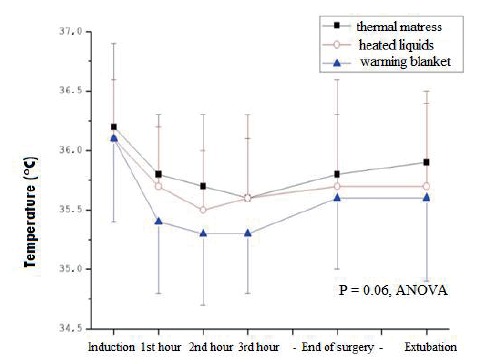
Mean value of esophageal temperature between groups and times studied. Campinas, SP, Brazil, 2015

## Discussion

The incidence of pathologies that require resectability of organs of the gastroenteric tract has increased regardless of gender. The gender variable did not demonstrate homogeneity, revealing a greater number of women randomized to this study. This fact may be a consequence of a higher male mortality, if observed in both absolute numbers and addressing coefficients and their causes. Male mortality coefficients are higher at all ages.

Therefore, in the studied age range, there are more females than males because mortality is higher in the second group^14^.

In this way, there is the need to adopt heating strategies for the prevention of mortality. 

Several studies comparing heating methods have been published, and there is great differences among them on which would be the best heating method to guarantee normothermia in the intraoperative period^6^ ^,^ ^15^. The implementation of interventional measures, such as the preheating of all patients with hot air systems with an overlap blanket in the 15 minutes that preceded their referral to the operating room, and the use of blankets until the beginning of anesthetic induction, is essential in the prevention and internal redistribution of heat in the body, the main cause of perioperative hypothermia. This method increases the heat content of the peripheral compartment of the organism, causing a reduction in the temperature gradient between the central and peripheral compartments. In this study, the esophageal temperature^14^ ^,^ ^16^ was monitored. A systematic review showed the need of 15 to 60 minutes of preheating, which prevented hypothermia^15^. In the present study, despite preheating, there was a drop in the patient’s temperature in the first three hours, and there was no complete recovery of temperature at the end of the intraoperative period in all the methods used. These results reinforce the adoption of the prevention measures proposed in several studies^13^ ^-^ ^19^. Following recommendations from the American Society of PeriAnesthesia Nurses (ASPAN)^13^, the temperature of the operating room (OR) should be maintained between 20 and 24ºC. In the present research, the mean temperature of the OR was between 22.5° C and 23.8°C, that is, within the range established and recommended by ASPAN.

Other authors suggest that the use of plastic and metalized blankets is of little use in preventing intraoperative heat loss, so it is necessary to use active systems to maintain patient normothermia. In these studies, the tympanic temperature was measured, always using the same thermometer, at different moments, at the entrance of the room and after anesthetic induction^18^ ^-^ ^19^.

Active heating had better results, mainly through the forced-air warming blanket, maintaining the body temperature close to normotermia^8^.

The tympanic thermometer was used to measure the effectiveness of the use or not of blankets in surgeries of the elderly^6^.

In the present study, we measured the temperatures with an oesophageal thermometer and there was no significant difference between the measurements in the studied groups. 

In the present survey, despite the use of active heating methods, there was a decrease in temperature rather than recovery at the end of the procedure in all groups. In a study performed with forced air heaters, there was reduction of heat loss if placed under the patient, allowing circulation around, resulting in loss of heat by irradiation convection^20^, although some authors state that forced air heating is proved to be very effective, and when it is associated with the room temperature adjustment of the operating room it contributes to the prevention of perioperative hypothermia^20^ ^-^ ^22^. We observed, in the present study, that the use of the underbody warming blanket did not prevent intraoperative hypothermia. 

In the present study, there was no significant difference between the three methods used. The literature reinforces the need for the concomitant use of intravenous fluid with measures of heat conservation, since they presented a significant reduction of the accidental incidence of preoperative hypothermia in gynecological and abdominal surgeries, as well as associated complications during orthopedic procedures^21^. 

Severe hypothermia tends to occur more frequently in long-term surgeries, including the abdominal and thoracic ones, and especially those with a time greater than 180 minutes. In this study, the esophageal temperature was measured, which demonstrates accuracy of measurement^9^ ^,^ ^21^. The mean surgical time was greater than 120 minutes with a median of 285 minutes.

The losses and the volumes administered are related to the longer ICU time and hospitalization^9^. The mean of these variables was homogeneous between the groups, compared to the literature, for these procedures.

A meta-analysis has shown that, on average, there is a decrease in body temperature by 1.5° C during the intraoperative period, increasing hospital costs in US$ 2,500 to US$ 7,000 per surgical patient^11^. 

We highlight, as relevant aspects of this study, the prevention of intraoperative hypothermia and the nursing care that should be provided to patients in this period in order to reduce the occurrence of hypothermia. The perioperative nurse is the most qualified professional to evaluate the most suitable heating method for each surgical procedure. In addition, it is crucial that a university hospital, where high complexity procedures are performed, has several active heating options that meet the needs of patients.

## Limitations of the present study

In the literature, reports of hypothermia are frequent, possibly secondary to anesthetic procedures, room temperature and surgical time. In this study, the occurrence of mild hypothermia throughout the intraoperative period was evidenced, despite all the precautions for pre-heating in the preoperative period. 

It is also vitally important to carry out new prospective studies using multicenter studies for the external validation of the evidences observed here. These are essential prerequisites for skilled nursing care and patient safety assurance.

## Conclusion

There was no statistically significant difference related to the effectiveness between the three active heating methods used in the prevention of intraoperative hypothermia in open gastroenterological surgeries.

Given the results evidenced in the present study, we concluded that all patients presented mild hypothermia, not recovering the temperature of entrance in the operating room, regardless of the method used.
